# The use of three implants to support a fixed prosthesis in the management of the edentulous mandible: a systematic review

**DOI:** 10.1186/s40729-022-00423-5

**Published:** 2022-06-17

**Authors:** Murtaza Hirani, Maria Devine, Olamide Obisesan, Cathy Bryant

**Affiliations:** 1 Department of Oral Surgery, King’s College Dental Hospital, Bassemer Road, London, SE5 9RS UK; 2 Department of Oral Surgery, Eastman Dental Hospital, Huntley Street, London, WC1E 6DG UK

**Keywords:** Dental implant, Fixed prosthesis, Edentulous mandible, Survival rate, Marginal bone loss, Patient satisfaction, Systematic review

## Abstract

**Background:**

Dental implants have been widely utilised as a treatment modality for prosthetic rehabilitation. The aim of this study was to evaluate the implant and prosthetic survival rate, changes in marginal bone level, and patient satisfaction outcomes with the use of three implants to support a fixed prosthesis in the edentulous mandible.

**Methods:**

A comprehensive electronic search was performed in the MEDLINE, Embase and Cochrane databases to retrieve studies that met the selection criteria. Sixteen articles were selected which consisted of two randomised controlled trials, eight prospective cohort studies, five retrospective studies and one case series.

**Results:**

A total of 2055 implants were placed in 685 patients with a mean age of 62.2 years. The mean cumulative implant survival rate was 96.2% over a mean follow-up period of 3.35 years. Mean marginal bone loss recorded was 1.25 mm and high patient satisfaction rates were reported across the studies.

**Conclusion:**

The use of three implants to support a fixed prosthesis appears to be a successful approach to restoring the edentulous mandible in the short-to-medium term. Further longitudinal comparative studies are required to support longer-term success, and to guide minimum implant dimension requirements for the technique.

## Background

Since the evolvement of osseointegrated implants, rehabilitation of the edentulous mandible has often proved clinically challenging. Following exodontia, resorption in the mandible usually affects the two coronal thirds with the basal third remaining stable. These anatomical considerations significantly influence the implant and prosthetic parameters required to achieve primary implant stability. The use of four or more implants to support a fixed prosthesis in the edentulous mandible is well documented with high levels of clinical outcomes recorded [1, 2]. Despite this predictability, the use of a three-implant supported fixed prosthesis offers the potential to deliver a more cost-effective method of oral rehabilitation in the lower arch; an important consideration given that edentulism is most prevalent in low-income subpopulations [3].

One of the earliest techniques that aimed to reduce the number of implants and achieve osseointegration by immediate loading in the edentulous mandible was the Brånemark Novum^®^ (Nobel Biocare, Gothenburg, Sweden) protocol. The premise behind the technique was the placement of three implants in the anterior region of the mandible. Despite its potential, there were a number of prerequisites such as requirements for a minimum dimension of mandibular height, uniform bone thickness and specific anatomical relationships that were compatible with the prefabricated components. Moreover, it was sensitive to the angulation of implants and relied on the acquisition of surgical guides designed specifically for its use and as a result, the system was eventually withdrawn from commercial use.

Despite these shortcomings, the concept demonstrated many favourable clinical outcomes [4]. This inspired modifications of the technique in which three conventional and prefabricated prosthetic components were used for immediately loading a fixed prosthesis in the mandible connected by a metal infrastructure. These newer concepts of prosthetic rehabilitation were designed with much less reliance on prefabricated drilling guides and specific instrumentation, thereby facilitating their use for a wider population. Their use demonstrated benefits similar to those of the original Novum protocol, in reducing both surgery time and costs, with the aim of improving patient comfort and satisfaction.

Although the concept of using three implants to support a lower fixed prosthesis is no longer novel, there has been a resurgence in interest surrounding this technique using modern bridge substructures, such as the recently introduced Trefoil^®^ (Nobel Biocare, Gothenburg, Sweden) system, which claims to offer a definitive standardised prosthetic solution [5]. With an increase in popularity of such techniques, it is important to understand the limitations of these systems and also their performance in relation to more established techniques. This systematic review aimed to evaluate the implant and prosthetic survival rate, changes in marginal bone level, and patient satisfaction associated with a three-implant supported fixed prosthesis for rehabilitation of the edentulous mandible over a follow-up period of at least 1 year.

## Materials and methods

### Registry protocol

The systematic review was conducted according to the Preferred Reporting Items for Systematic Reviews and Meta-Analyses (PRISMA) statement [6]. The protocol for this study was registered in the International Prospective Register of Systematic Review (PROSPERO) database, CRD 42019159711. The focused research question being addressed was ‘What is the implant and prosthetic survival rate, changes in marginal bone level and patient satisfaction outcomes with the use of three implants to support a fixed prosthesis in the edentulous mandible?’.

This was developed according to the PICO framework for evidenced-based practice [7]. The population (P) comprised of adult participants with an edentulous mandible undergoing implant treatment; the intervention (I) was three implants to support a lower fixed prosthesis; the comparison (C) were mandibular fixed complete prosthesis with more than three implants; the outcomes (O) measured were implant and prosthetic survival rate, marginal bone loss, and patient satisfaction.

### Search strategy

An electronic literature search was conducted using the following databases: MEDLINE, Embase, The Cochrane Oral Health's Trials Register and The Cochrane Central Register of Controlled Trials, (CENTRAL) up until January 2022. The search strategy was formulated using a combination of medical subject heading (MeSH) terms and free text terms that included: “three implant” OR “fixed prosthesis” OR “immediate rehabilitation” AND “edentulous mandible. These terms were tailored according to the requirement of each electronic database. This was supplemented by a manual search including the bibliographies of all articles selected for screening, as well as from previous published reviews of relevance. The search was limited to articles published in the English language.

### Eligibility criteria

The inclusion criteria for selecting articles were: case series, retrospective and prospective studies, and randomised controlled trials reporting on human subjects with a minimum follow-up period of 1 year. Studies were included that reported on the use of three-implant supported fixed prostheses for rehabilitation of the edentulous mandible in adult participants. Outcomes were recorded from studies evaluating the implant and prosthetic survival rate, marginal bone loss and patient satisfaction. The following exclusion criteria were applied: animal studies, in vitro studies, case reports, commentaries and letters of correspondence, in addition to any studies with insufficient data reporting on the outcome measures of interest.

### Study selection

The electronic database search was carried out by the first review author (MH) as part of the data collection process. The full search results from all databases were exported to EndNote^®^ reference manager software (Clarivate Analytics, Philadelphia, USA) to remove duplicates, and the title and abstract of each remaining article were screened individually. This process was repeated independently by the second reviewer (MD) and any disagreement in inclusion of titles/abstracts was resolved by discussion or contact with a third reviewer (CB). In the second review phase of the study, the complete texts of the articles selected were read by two reviewers (MH, MD) to assess eligibility for inclusion. Any differences in the selection of studies were resolved by discussion with a third reviewer (CB).

### Data extraction

The data extraction was undertaken independently by two reviewers (MH and MD) using a standardised data collection form to ensure systematic recording of the outcome measures. The information was processed and tabulated to produce a summary of the findings using Microsoft Excel^®^ (Microsoft Corporation, Washington, USA). The information extracted included the study design and population, participant demographics, observation period, loading protocol and the number of implants placed. In addition, the relevant characteristics of the outcome measures were recorded including implant and prosthetic success, marginal bone loss and patient satisfaction. If the articles included were missing any relevant information, or in the event of any ambiguity, the corresponding authors were contacted by e-mail for further clarification. Results of data extraction were compared and any disagreements resolved by further analysis of the relevant article and agreement between the reviewers.

### Quality assessment

The quality of the included studies was evaluated according to the study design. Two randomised controlled trials identified using the search strategy were assessed using the Cochrane Risk of Bias (RoB 2) tool [8]. The remaining non-randomised studies included were assessed using the Cochrane Risk Of Bias In Non-randomised Studies (ROBINS-I) tool [9].

### Statistical analysis

Following a preliminary evaluation of the selected studies, considerable heterogeneity was found to be present in the study designs, outcome measures, and results derived. As a result, it was not possible to perform valid meta-analysis to calculate adjusted pool estimates, and a descriptive synthesis of the data is presented. Mean values and standard deviations (SD) were calculated using SPSS^®^ (IBM Corporation, New York, USA), and the level of statistical significance across all comparative studies described was set at *P* < 0.05.

## Results

### Search results

**Fig. 1:**
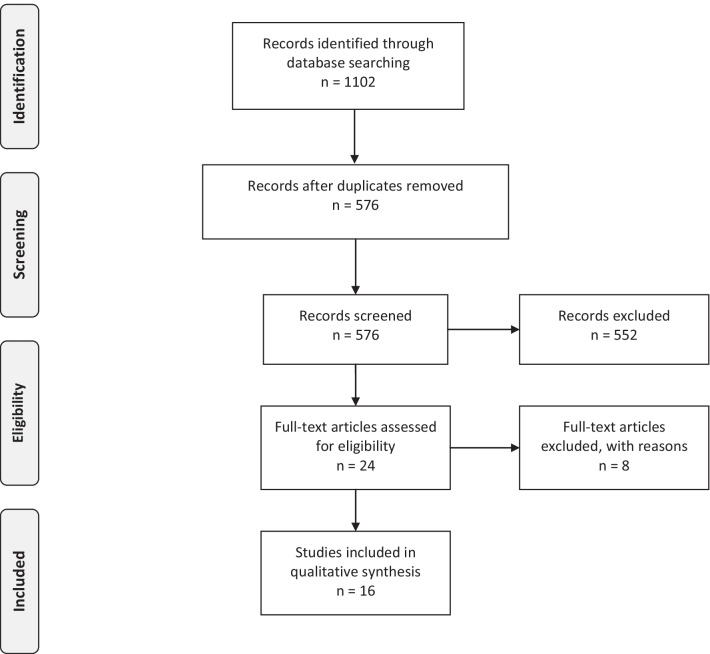
PRISMA flow diagram of the literature search

The electronic search yielded a total of 1102 articles including 458 from MEDLINE, 402 from Embase and 242 from the Cochrane databases. Following the removal of duplicate articles, 576 titles and abstracts were read with 24 articles qualifying for full text retrieval. After further screening, 8 articles were excluded that did not meet the eligibility criteria. As a result, 16 articles were included in the final review published between 2001 and 2022 [10–25]. The results of the search strategy are outlined in the PRISMA flow diagram (Fig. 1). Details of the studies with the main findings are summarised in Tables 1 and 2. The reasons for exclusion of studies are given in Table 3 [26–33].

**Table 1:** Characteristics of the included studies

Year	First Author	Study design	Patients	Implants	Mean age years	Mean follow up years	Follow up range months	Implant survival	Prosthetic survival	Marginal bone loss	Loading	System
2001	De Bruyn [10]	Prospective	20 (8M, 12F)	60 Straight	64 (41-80)	3	36	90.5%	85%	2.1 mm	Immediate	Brånemark®
2003	Engstrand [11]	Prospective	95 (53M, 42F)	285 Straight	68.5 (45-89)	2.5	12-60	93.3%	99%	0.73 mm	Immediate	Brånemark Novum®
2003	Henry [12]	Prospective	51 (28M, 23F)	153 Straight	62 (43-79)	1	12	91%	94%	0.4 mm	Immediate	Brånemark Novum®
2004	Van Steenberghe [13]	Prospective	50 (25M, 25F)	150 Straight	57 (45-80)	1	12	92.7%	95%	1.1 mm	Immediate	Brånemark Novum®
2009	Gualini [14]	Retrospective	15 (11M, 4F)	45 Straight	64 (55-78)	5	60	91%	87%	NR	Immediate	Brånemark Novum®
2011	De Kok [15]	Prospective RCT	10 (5M, 5F)	30 Distal tilted	62 (47-76)	1	12	100%	100%	NR	Delayed	Astra Tech Osseospeed®
2011	Hatano [16]	Retrospective	132 (65M, 67F)	396 Distal tilted	63 (35-85)	5	12-132	96.7%	92.4%	NR	Immediate	Brånemark®
2012	Oliva [17]	Retrospective	12 (8M, 4F)	36 Straight	49 (39-79)	5	60	100%	100%	NR	Delayed	Straumann®/Osstem®
2012	Rivaldo [18]	Prospective	33 (12M, 21F)	99 Straight	NR (38-83)	1.5	18	97.8%	NR	0.8 mm	Immediate	NR
2018	Beresford [19]	Prospective	12 (5M, 7F)	36 Straight	69 (60-81)	1	12	100%	100%	NR	Immediate	Nobel Biocare®
2018	Cannizzaro [20]	Prospective RCT	12 (NR)	36 Straight	57.5 (39-78)	1	12	91.7%	97.2%	0.22 mm	Immediate	Sweden & Martina®
2018	Primo [21]	Prospective	20 (4M, 16F)	60 Distal tilted	64 (46-81)	1.5	18	98.3%	100%	1.75 mm	Immediate	Brånemark Novum®
			22 (4M, 18F)	66 Distal tilted	71 (57-83)	1.5	18	98.5%	100%	1.75 mm	Delayed	Brånemark Novum®
2019	Menini [22]	Case Series	4 (3M, 1F)	12 Straight	54 (41-65)	16	192	100%	100%	2.2 mm	Immediate	Brånemark Novum®
2019	Mezzari [23]	Retrospective	58 (23M, 35F)	174 NR	63 (46-61)	5	60	97.1%	93.1%	2.29 mm	Immediate	Brånemark Novum®
2020	Anya [24]	Retrospective	29 (16M, 13F)	87 Distal tilted	65 (NR)	6	72	100%	NR	1.0 mm	Immediate	Hi-Tec Logic Plus®
2020	Higuchi [25]	Prospective	110 (50M, 60F)	330 Straight	62 (NR)	1	12	97.5%	97.3%	0.62 mm	Immediate/Early	Nobel Biocare Trefoil®

*NR* not reported

**Table 2:** Overview of the implant and prosthetic failures

Year	First author	Study design	Number implants	Implant failures	Number prosthesis	Prosthetic failures	Prosthetic complications
2001	De Bruyn [10]	Prospective	60	6	20	3	2
2003	Engstrand [11]	Prospective	285	18	95	1	17
2003	Henry [12]	Prospective	153	14	51	3	15
2004	Van Steenberghe [13]	Prospective	150	11	50	NR	NR
2009	Gualini [14]	Retrospective	45	4	15	2	24
2011	De Kok [15]	Prospective RCT	30	0	10	0	32
2011	Hatano [16]	Retrospective	396	13	132	NR	NR
2012	Oliva [17]	Retrospective	36	0	12	0	2
2012	Rivaldo [18]	Prospective	99	2	33	NR	NR
2018	Beresford [19]	Prospective	36	0	12	0	5
2018	Cannizzaro [20]	Prospective RCT	36	3	12	1	1
2018	Primo [21]	Prospective	60	1	20	0	10
			66	1	22	0	10
2019	Menini [22]	Case series	12	0	4	0	2
2019	Mezzari [23]	Retrospective	174	5	58	NR	27
2020	Anya [24]	Retrospective	87	0	29	NR	10
2020	Higuchi [25]	Prospective	330	8	110	NR	42
Total			2055	86	685	10	199

*NR* not reported

**Table 3:** Excluded studies

Year	First author	Study design	Reason for exclusion
1999	Brånemark [26]	Prospective	Patient sample reported by same group in a further included article [13]
2001	Chow [27]	Prospective	Patient sample receiving more than three implants in mandible
2003	Hatano [28]	Prospective	Patient sample reported by same group in a further included article [18]
2003	Krug [29]	Prospective	Insufficient duration of follow-up
2003	Popper [30]	Prospective	Insufficient duration of follow-up
2005	Abarca [31]	Retrospective	Insufficient data for outcomes measures
2013	Yi [32]	Retrospective	Patient sample receiving implants in partially edentulous mandibles
2015	Tealdo [33]	Retrospective	Patient sample reported by same group in a further included article [24]

### Study characteristics

From the 16 articles identified for inclusion, two were randomised controlled trials [15, 20], eight prospective cohort studies [10–13, 18, 19, 21, 25], five retrospective studies [14, 16, 17, 23, 24] and one was a case series [22]. Five studies were conducted in private practice [14, 16, 17, 19, 20], four within university dental departments [11, 13, 15, 22], four in specialised implant institutes [18, 21, 23, 24] and three multicentre studies [10, 12, 25]. The eligible studies included a total of 685 patients that had 2055 implants placed. Eleven of the studies placed straight implants [10–14, 17–20, 22, 25], four studies had distally tiled implants [15, 16, 21, 24] and one study did not report on the implant angulation [23], with the majority employing the Brånemark Novum^®^ system. The subjects included 320 males and 353 females with one study not reporting gender for the cohort of patients analysed. The age ranged from 35–89 years with a mean of 62.2 years (SD 5.62) and the duration of follow-up was from 1–16 years. The quality of the studies as assessed using the Cochrane risk of bias tools is included in Tables 4 and 5.

**Table 4:** Cochrane risk of bias (RoB 2) tool for randomised controlled trials

Year	First Author	Study design	Random sequence generation (selection bias)	Blinding of participants and personnel (Performance bias)	Blinding of outcome assessment (Detection bias)	Incomplete outcome data (Attrition bias)	Selection reporting (Reporting Bias)	Other sources of bias	Overall risk of bias
2011	De Kok [15]	Prospective RCT	Some concerns	Some concerns	Some concerns	Low risk	Low risk	None	Some concerns
2018	Cannizzaro [20]	Prospective RCT	High risk	High risk	Some concerns	Low risk	Low risk	None	High risk

**Table 5:** Cochrane risk of bias (Robins-I) tool for non-randomised studies

Year	First author	Study design	Confounding	Selection of participants	Classification of interventions	Deviation from interventions	Missing data	Measurement of outcomes	Selection of results	Overall
2001	De Bruyn [10]	Prospective	Moderate	Low	Low	Low	Low	Low	Low	Moderate
2003	Engstrand [11]	Prospective	Moderate	Moderate	Low	Moderate	Moderate	Low	Moderate	Moderate
2003	Henry [12]	Prospective	Moderate	Low	Moderate	Moderate	Moderate	Moderate	Low	Moderate
2004	Van Steenberghe [13]	Prospective	Moderate	Moderate	Low	Moderate	Moderate	Moderate	Moderate	Moderate
2009	Gualini [14]	Retrospective	Moderate	Moderate	Moderate	Moderate	Moderate	Moderate	Low	Moderate
2011	Hatano [16]	Retrospective	Moderate	Moderate	Moderate	Moderate	Moderate	Moderate	Moderate	Moderate
2012	Olivia [17]	Retrospective	Moderate	Moderate	Serious	Moderate	Serious	Moderate	Moderate	Serious
2012	Rivaldo [18]	Prospective	Moderate	Moderate	Moderate	Moderate	Low	Moderate	Low	Moderate
2018	Beresford [19]	Prospective	Moderate	Moderate	Moderate	Moderate	Low	Moderate	Moderate	Moderate
2018	Primo [21]	Prospective	Moderate	Moderate	Moderate	Moderate	Low	Moderate	Low	Moderate
2019	Menini [22]	Case series	Moderate	Serious	Moderate	Moderate	Low	Low	Moderate	Serious
2019	Mezzari [23]	Retrospective	Moderate	Moderate	Moderate	Moderate	Low	Moderate	Low	Moderate
2020	Anya [24]	Retrospective	Moderate	Moderate	Moderate	Moderate	Low	Moderate	Moderate	Moderate
2020	Higuchi [25]	Prospective	Moderate	Moderate	Low	Moderate	Low	Moderate	Low	Moderate

### Implant survival rate

All studies included in this review reported implant survival rates. Survival ranged from 90.5 to 100% with a mean cumulative survival rate of 96.2% (SD 3.67) at a mean follow-up period of 3.35 years. Implant success was initially defined as the presence of a functional implant in the absence of clinical signs of pain, suppuration, or mobility with no radiographic features of failed osseointegration [34]. From the 2055 implants placed, 86 failures were recorded, representing a rate of 4.2%, across the studies.

Immediate loading has been described as taking place within 1 week after implantation, early loading from 1 week up to 2 months, and delayed loading from 2 months onwards [35]. Twelve studies analysed the immediate loading of implants alone [10–14, 16, 18–20, 22–24], two studies employed an delayed loading protocol [15, 17], and one study performed both immediate and early loading without differentiation of the outcome measures between the two [25]. The final comparative study assessed the clinical and radiographic outcomes of an immediate versus delayed loading approach and found no statistically significant difference (*P* > 0.05) in implant survival rate between the two loading protocols [21].

### Prosthetic survival rate

Prosthetic survival rate was recorded in all studies apart from two [18, 24]. Survival ranged from 85 to 100% with a mean cumulative prosthetic survival rate of 96% (SD 4.9) at the end of the follow-up period. A prosthetic construction was considered successful if in continuous and unrestricted function, and stable upon manual clinical examination [34]. Following the placement of 685 fixed prosthesis, only 10 absolute prosthetic failures were recorded. However all studies reporting the cause of prosthetic failure, attributed this to being a direct result of implant failure [10–12, 14, 20].

Three studies did not report on prosthetic complications that occurred following treatment [13, 16, 18]. The most commonly recorded complications included screw loosening, resin or acrylic tooth fractures and occlusal adjustments that were required. Overall, 199 complications in total were recorded across the studies, although none of these resulted in the failure of the fixed prosthesis and overall treatment outcome.

### Marginal bone loss

Changes in marginal bone levels were evaluated in 11 of the included studies [10–13, 18, 20–25]. In all studies, serial intra-oral periapical [10–13, 20, 22, 25] or panoramic radiographs [18, 21, 23, 24] were used to calculate bone-level changes using a fixed reference point on the implant fixture in relation to the alveolar bone crest. Marginal bone loss ranged from 0.22 to 2.29 mm over a follow-up period of 1–16 years. For studies that had measured marginal bone loss at each of the three implant sites (right, centre and left), an average was calculated across the values recorded and the mean marginal bone loss around the implants evaluated was 1.25 mm (SD 0.74). Implant success has also been defined as bone loss of up to 1.5 mm in the 1st year and up to 0.2 mm per year in subsequent years (2.3 mm after 5 years) [34]. Using this criterion, nine studies were within the range considered indicative of implant success [11–13, 18, 20, 22–25].

### Patient satisfaction

Eight studies in total included patient satisfaction outcomes that were assessed by means of questionnaire [10, 12, 15, 16, 19, 24, 25]. Overall satisfaction with the treatment result ranged from 77 to 100% over a follow-up period from 1–6 years. Two studies assessed both patient satisfaction and oral health-related quality of life for a three-implant supported fixed dental prosthesis in the mandible in comparison to a conventional mandibular removable dental prosthesis [15, 19]. Patient satisfaction was evaluated using a visual analogue scale (VAS) and oral health-related quality of life measured using a modified version of the oral health impact profile (OHIP). The studies found that both treatment modalities provided a similar improvement in the assessment tools when compared with pretreatment scores. There was found to be no statistically significant difference (*P* > 0.05) observed between the two groups, although the mean overall scores were higher for fixed prostheses.

## Discussion

The purpose of this systematic review was to evaluate the implant and prosthetic survival rate, changes in marginal bone level, and patient satisfaction with the use of three implants to support a fixed prosthesis in the management of the edentulous mandible. There was found to be limited high-quality evidence with only two randomised controlled trials identified [15, 20]. One of these studies was considered to have some concern of risk of bias [15], whilst the other was deemed to be at high risk [20]. The quality of evidence and risk of bias of the remaining non-randomised studies were assessed using the Cochrane Risk of Bias (RoB 2) assessment tool to reduce the risk of subjective evaluation, and were classified as ranging from moderate to severe. Therefore, a degree of caution would be advocated with interpretation of the results.

To the author’s knowledge, there have been two previous systematic reviews conducted assessing outcomes with fixed mandibular prostheses supported by three implants [36, 37]. The reviews concluded similar limitations, particularly the heterogeneity of the studies analysed and the lack of high-level evidence studies comparing two groups of three implants with larger numbers of implants. The strength of this review was the inclusion of two prospective randomised clinical trials. Moreover, challenges included obtaining outcomes for longer term follow-up periods with the longest duration previously assessed at 6 years. There was also inclusion of research conducted with a significantly longer follow-up period of 16 years that confirmed favourable outcomes particularly in relation to both implant and prosthetic surgical rates.

This review revealed a mean cumulative implant survival rate of 96.2% (SD 3.67), over a mean follow-up period of 3.35 years. This was found to be comparable to previous systematic reviews reporting survival rates ranging from 98.3 to 99.2% for the use of four implants supporting a fixed prosthesis in the edentulous mandible over a similar time period [38, 39]. The study that included the longest period of follow-up, 16 years, reported a survival rate of 100% using the Novum protocol [22]. The main limitation of this case series was the small sample size included, reducing the external validity of the findings. The study reporting the lowest implant survival rate, 90.5%, had a follow-up period of 3 years [10]. This involved an early loading protocol and the authors attributed the higher failure rate to increased compressive forces on the components, leading to technical failures of the implants.

Similar outcomes were found in relation to prosthetic survival rates across the studies. A mean cumulative prosthetic survival rate of 96% (SD 4.9) was recorded with prosthetic failures related to implant loss. Although a relatively high survival rate was reported, limitations of this technique involving the placement of three implants were highlighted by the fact that failure of one implant resulted in the prosthesis being condemned. Although the use of a fewer number of implants can be more of a financially viable option for patients, the costs involved in potential prosthetic reconstruction need to be analysed with further research in comparison to systems employing a greater number of implants. Prosthetic complications were not recorded in all studies and there was found to be a lack of uniformity in the manner of their reporting. The findings were similar to previous systematic reviews concluding that although complications were infrequently reported, they were not shown to influence the overall survival of the prosthesis [40, 41].

Based on the studies included in this review, the cumulative mean marginal bone loss recorded was 1.25 mm (SD 0.74). The majority of peri-implant marginal bone loss occurred during the 1st year following prosthetic loading, in keeping with previously published meta-analysis [42]. Since implant success was initially defined in relation to peri-implant bone resorption [34], there has been a lack of consensus for this measure with various other proposals suggested [43, 44]. Only five studies alluded to this success criterion when evaluating marginal bone level changes [10, 11, 18, 21, 23]. Limitations in assessment included an absence of standardised methods used for radiographic measurements, with only one study reporting detailed calibration for periapical radiographs [19]. Thus, studies performing analysis by means of panoramic radiographs would have had lower precision, as a result of challenges involved with assessment of the anterior region of the mandible [45].

A single study reviewed compared the clinical and radiographic outcomes of immediate versus delayed loading [21]. This study failed to find any statistically significant association (*P* > 0.05) for implant survival or peri-implant bone loss with loading protocol. Overall this finding concurred with previous systematic reviews conducted to determine the influence of various loading times on fixed prostheses supported by greater than three implants, which found no significant impact on either implant survival or marginal bone loss [34]. Across the studies, there was found to be one randomised controlled trial that compared outcomes for the immediate loading of three versus four implants supporting fixed prosthesis [20]. This highlighted the scope for further research required within this area. Statistical analysis was performed using Fisher’s exact probability test, independent-sample *t*-tests, and analysis of covariance. The study concluded no statistical significant differences (*P* > 0.05) for implant survival and prosthetic survival, complications, and marginal peri-implant bone levels between the two groups. Despite this, the preliminary results from this short-term follow-up study of 1 year, combined with the higher risk of bias, would preclude any significant clinical recommendation for the protocols described.

Patient-centred outcomes have been recognised as important determinants in implant success [46, 47]. Although a number of scoring methods were used such as visual analogue scales (VAS) and oral health impact profile (OHIP) questionnaires, the need for standardised reporting of patient-centred outcomes was further emphasised [48]. In two studies, patient satisfaction levels were reported as being similar to those rehabilitated with two-implant retained mandibular removable prosthesis, with no significant difference (*P* > 0.05) between both groups [15, 19]. These results supported the use of a three–implant retained fixed prosthesis as an alternative option in improving these parameters with this cohort of patients.

Previous systematic reviews have aimed to determine the optimal number of implants supporting complete-arch fixed-dental prostheses in both the maxilla and mandible [49, 50]. The strength of this study was within the methodology of clearly reporting on papers focusing on the use of three implants to support a fixed prosthesis in the edentulous mandible with evaluation of multiple outcome measures. As a result, there were a smaller number of studies selected due to the strict inclusion criteria applied. The main limitation of this report was the low level of evidence available with only two randomised controlled trials included, and the relatively short mean observational period that could influence survival rates. Moreover, it was not possible to evaluate results to include additional variables in implant dimensions, as well as angulation and distribution within the arch, which are valuable treatment regulators that can guide loading protocols.

There were numerous confounding factors that may have directly impacted treatment outcomes such as investigators placing implants of different brands and surface treatments. Nevertheless, it is considered that the results extracted from this review can be useful to practitioners. Newer systems and designs are being released that may further provide higher survival rates, although there was found to be limited evidence available at present with only one clinical study identified within this review. Further research is necessary that is focused on three-implant supported design systems that directly compare outcomes with different number of implants and loading protocols, to validate this technique and consolidate its use in routine clinical practice.

## Conclusion

In conclusion, current evidence suggests that a three-implant supported fixed prosthesis for the edentulous mandible is a successful treatment strategy presenting high implant and prosthetic survival rates over the short-to-medium term. Further well-designed controlled clinical trials are required to evaluate longer-term outcomes, with supplemental data correlating implant dimensions and prosthetic design.

## Data Availability

All data generated or analysed during this study are included in this published article.
